# Nutritional Rehabilitation in Patients with Malnutrition Due to Crohn’s Disease

**DOI:** 10.3390/nu11122947

**Published:** 2019-12-04

**Authors:** Lidia Santarpia, Lucia Alfonsi, Fabiana Castiglione, Maria Carmen Pagano, Iolanda Cioffi, Antonio Rispo, Maurizio Sodo, Franco Contaldo, Fabrizio Pasanisi

**Affiliations:** 1 Internal Medicine and Clinical Nutrition Unit, Department of Clinical Medicine and Surgery, Federico II University Hospital of Naples, Via Pansini, 5, 80131 Naples, Italy; lucia.alfonsi@unina.it (L.A.); carmenpag@libero.it (M.C.P.); iolanda.cioffi@unina.it (I.C.); contaldo@unina.it (F.C.); pasanisi@unina.it (F.P.); 2 Gastroenterology Unit, Department of Clinical Medicine and Surgery, Federico II University Hospital of Naples, Via Pansini, 5, 80131 Naples, Italy; fabiana.castiglione@unina.it (F.C.); antoniorispo@email.it (A.R.); 3 General and Transplantation Surgery, Department of Public Health, Federico II University Hospital, 80131 Naples, Italy; maurizio.sodo@unina.it

**Keywords:** crohn’s disease, malabsorption, malnutrition, nutritional rehabilitation

## Abstract

Background: Crohn’s disease (CD) is a chronic inflammatory bowel disease frequently associated with malabsorption and secondary protein-energy malnutrition (PEM). Methods: Biochemical and clinical data of 63 (34 females, 29 males) patients with PEM due to CD sent to our outpatient unit for nutritional evaluation were retrospectively analyzed. Patients were divided into two groups, according to disease activity. Thirty-eight patients (group A) had the active disease, and 25 patients (group B) suffered from malabsorption resulting from past intestinal resections due to CD. After a physical and hemato-biochemical evaluation at the first visit, all patients received disease-specific personalized dietetic indications. When indicated, oral nutritional supplements, oral/parenteral vitamins, micronutrients, and electrolytes, up to parenteral nutrition, were prescribed. Results: After 1, 3, and 6 months of nutritional therapy, body weight, body mass index (BMI), and serum butyryl-cholinesterase significantly improved in both groups. In 8 out of 13 (61.5%) patients with a cutaneous stoma, intestinal continuity was restored. Conclusions: This study confirms the effectiveness of nutritional rehabilitation and provides information on the time required for nutritional treatment in patients with CD, both during the acute phase and after malabsorption due to intestinal resection.

## 1. Introduction

Crohn’s disease (CD) is a chronic inflammatory bowel disease that can involve all segments of the gastrointestinal tract. During the acute phases of the disease, malabsorption and catabolic effects of acute inflammation cause weight loss and malnutrition with an incidence of 25–80% [1,2,3,4].

Patients with CD can develop chronic intestinal failure (CIF) as a result of reduced absorption due to the inflammatory involvement of intestinal mucosa, mechanical obstruction, or wide intestinal resections. These factors may act alone or in combination, impairing the ability of the gut to maintain protein-energy, fluid, electrolyte, or micronutrient balance, particularly in the presence of a stoma (jejuno or ileostomy) [5,6].

Documented malnutrition in patients with Inflammatory Bowel Disesses should be treated appropriately, as it worsens the prognosis, complication rates, mortality, and quality of life [7].

For these reasons, CD patients often require personalized dietetic advice, oral or parenteral macro-and micro-nutrients, and fluid and electrolyte supplementation, according to their clinical and nutritional conditions, as well as their absorptive capacity [3].

The study evaluates the types, modalities, and durations of nutritional interventions in patients with malnutrition secondary to CD.

## 2. Patients and Methods

Biochemical and clinical data of 63 (34 F, 29 M) patients followed at a specialized outpatient unit for Clinical and Artificial Nutrition of Federico II University Hospital in Naples (Italy), from January 2016 to December 2017, were retrospectively analyzed.

At the first visit, accurate medical history, a physical exam with particular attention to nutritional status (e.g., habitual and actual body weight, and unintentional weight loss in the last 6 months), and anthropometric measures (weight, height, and body mass index (BMI)) were collected. A dietetic inquiry by a specialized dietitian was included.

Blood samples was taken for routine hemato-biochemical exams; in particular, albumin, transferrin, prealbumin, butyryl-cholinesterase (BChE), and lymphocyte count.

Clinically, disease severity was defined by the Crohn’s disease activity index (CDAI), consisting of eight variables: number of stools, presence and severity of abdominal pain, general well-being of the patient, presence of extra intestinal manifestations of disease, presence of abdominal masses, use of antidiarrheal drugs, and presence of anemia and weight loss. Patients with CDAI <150 were classified as being in the clinical remission phase, while patients with CDAI ≥150 were considered to have the active disease.

The patients were divided into two groups: Patients with malnutrition/malabsorption due to active disease (Group A) and patients in clinical remission with malnutrition/malabsorption due to intestinal resection (Group B). Malabsorption was testified by diarrhea, low nutritional indicators, weight loss, and remnant bowel length.

After baseline evaluation and according to their nutritional status, all patients received personalized dietetic advice.

In the case of protein energy malnutrition (PEM) and underweight condition, oral nutritional supplements (ONS) were prescribed for a supplementary intake of up to 250–300 kcal/day (1–1.5 kcal/mL, 20–30% proteins, 35–45% carbohydrates, 25–45% lipids, and fiber-, lactose-, and gluten-free), enriched with vitamins and micronutrients according to the RDA.

In the presence of protein malnutrition and normal to overweight, a modular protein powder (12 g/day) was chosen.

Hydro-electrolytic parenteral supplementation was generally necessary in the acute phase of CD with severe diarrhea and dehydration; parenteral nutrition was reserved for cases of associated malabsorption and malnutrition or during remission phases in patients with a short bowel and/or high-output intestinal stoma. The prescribed parenteral supply considered the patient’s nutritional status, oral intake, and losses; for guidance, the energy supply (expressed per kg body weight/day) was approximately 25–30 kcal (fluids, 25–30 mL; proteins, 1.2–1.5 up to 2.0 gr, unless the glomerular filtration rate (GFR) was <30 mL/min; and lipid/CHO ratio, 30/70% or 40/60% of the total energy supply), with vitamin and micronutrient enrichment according to the RDA [7].

Patients were followed up monthly or biweekly, according to their baseline clinical conditions.

For each patient group, baseline data were compared with evaluations at 1, 3, and 6 months.

The study was approved by the Ethics Committee of Federico II University (protocol number: 102/16).

## 3. Statistical Analysis

The data obtained were processed using a specific software for statistical analysis (SPSS ver. 15.0, IBM Corporation Inc., Chicago, IL, USA).

Values were expressed as the mean ± standard deviation (SD), median, min, and max. For the comparison between means, the T test was used for non-paired data, and the chi-squared test was used for categorical variables. Finally, analysis of variance (ANOVA) was used for the comparison among the groups. Differences were considered significant for *p* value < 0.05.

## 4. Results

The baseline characteristics of the 63 CD patients, subdivided into the two groups, are reported in Table 1. A total of 38 patients (19 F, 19 M; 42.1 ± 15.5 years) suffered from malabsorption/malnutrition due to mild/moderate activity disease (group A), and a total of 25 patients (15 F, 10 M; 40.9 ± 15.5 years) were in clinical remission with malabsorption due to intestinal resection (group B).

A total of 26 out of 38 (68.4%) patients in group A and 24 out of 25 (96.0%) patients in group B had undergone one or more intestinal resections, and 13 (20.6%) patients had an intestinal stoma—4 patients in group A (1 with a jejunostomy and 3 with ileostomies) and 9 patients in group B (4 with jejunostomies and 5 with ileostomies) (Table 2).

No differences emerged in the evaluated clinical and biochemical parameters between group A and B patients.

At the first visit, 12 out of 38 (31.6%) patients in group A and 11 out of 25 (44.0%) patients in group B had BMI ≤18.5 kg/m^2^. Additionally, 8 out of 38 (21.0%) patients in group A and 8 out of 25 (32.0%) patients in group B had lost at least 5%–10% of their body weight in the previous 6 months. Similarly, in both groups, the main hemato-biochemical exams were lower than normal values in a considerable percentage of patients (Table 2).

### 4.1. Dietetic Indications

All patients received detailed and personalized dietetic indications.

All dietary advice had the following characteristics [3,8,9]:

- Fiber-and lactose-free diet in the acute phase of the disease;
- Fiber was gradually reintroduced into the diet, based on individual tolerance, during the phase of slight/moderate activity;
- Milk and dairy products were gradually reintroduced in patients who did not report a pre-existing lactose intolerance before the acute phase onset; otherwise, lactose-free products were suggested, according to the individual’s tolerance.

### 4.2. Oral Supplements

Almost all patients received multivitamin and mineral tablets. A total of 21 out of 38 (55.3%) patients in group A and 11 out of 25 (44%) patients in group B received the prescription of a modular gluten -and lactose-free protein powder and/or ONS for a supplementary intake of up to 250–300 kcal/day (1–1.5 kcal/mL, 20–30% proteins, 35–45% carbohydrates, 25–45% lipids, and fiber-, lactose-, and gluten-free), enriched with vitamins and micronutrients according to the RDA.

### 4.3. Parenteral Therapy

Nine (23.7%) patients in group A needed parenteral (i.m. or i.v.) multivitamin complexes and intravenous electrolytes and micronutrients. In particular, i.m., B1–B6–B12 vitamins, folic acid, and vitamins A, D, and E were prescribed, following measurement of blood levels.

Rehydration therapy consisted of the prescription of intravenous electrolytic solutions (1–1.5 Lt × 3–7 days/week, containing K, Ca, P, and Mg according to the blood sample results). In addition, 9 out of 25 (36%) patients in group B received parenteral iron.

### 4.4. Home Parenteral Nutrition

Due to moderate to severe protein-energy malnutrition and severe malabsorption, 7 out of 38 (55.3%) patients in group A and 13 out of 25 (52%) patients in group B required parenteral nutrition: through peripheral venous access in 6, and through central venous access in 14. The prescribed parenteral supply considered the patient’s nutritional status, oral intake, and losses. For guidance, energy supply (expressed per kg body weight/day) was approximately 25–30 kcal (fluids, 25–30 mL; proteins, 1.2–1.5 up to 2.0 gr, unless GFR < 30 mL/min; and lipid/CHO ratio, 30%/70% or 40%/60% of total energy supply), with vitamins and micronutrients according to RDA.

The mean duration of therapy was 149 ± 236 (median 30; range 25–570) days for oral supplements, 45 ± 21 (median 45; range 30–60) days for hydration, and 203 ± 202 (median 90; range 30–730) days for Home Parenteral Nutrition (HPN).

### 4.5. Follow-Up Evaluations

As shown in Table 3 and Table 4, the nutritional intervention significantly influenced body weight, BMI, hemoglobin, and serum BChE in both groups. Other nutritional indicators also improved, but not significantly.

At present, all patients are still being followed up at our outpatient unit.

### 4.6. Patients with Intestinal Stomas

At baseline, 13 patients (20.6%) had an intestinal stoma: 5 had undergone jejunostomy and 8 had undergone ileostomy. All of these patients were referred to our outpatient unit soon after discharge from the surgical ward and, since then, they have all received home parenteral nutrition. In 8 out of 13 (61.5%) of these patients (4 with jejunostomies and 4 with ileostomies), intestinal continuity was restored after a median of 8 months (min 6, max 12) of HPN. In 5 out of 13 (38.5%) patients, the persistence of disease activity did not allow for surgical rehabilitation. The 8 rehabilitated patients were completely weaned off of HPN; among the 5 patients with a persisting stoma, 1 still received daily HPN, 2 received electrolytic infusions three times a week, and 2 (with ileostomies) were completely weaned off HPN due to intestinal adaptation.

## 5. Discussion

In this study, the effectiveness of nutritional intervention in a group of patients with malnutrition due to CD was retrospectively evaluated.

To this end, study patients were divided into two groups according to disease activity, the mechanisms responsible for malnutrition, and the resulting nutritional needs: a group of patients (Group A) with malnutrition/malabsorption due to active disease, and another group of patients (Group B) in clinical remission with malnutrition/malabsorption due to intestinal resection.

The type and modalities of the nutritional support were personalized—according to the patient’s clinical conditions (acute or chronic malabsorption, diarrhea, and presence of entero-cutaneous stomia)—and modulated during follow-up visits.

In each phase of the disease, appropriate dietary advice is an essential component of CD care: In the active phase, specific dietary indications may control the number and consistency of stools, consequently reducing dehydration, as well as electrolyte and nutrient losses [6].

Oral nutrition supplements (ONS) were prescribed as a minor supportive first-step therapy, in addition to normal food. Hydro-electrolytic parenteral supplementation was generally necessary in the acute phase of CD, with severe diarrhea and dehydration. Parenteral nutrition was reserved for cases of associated malabsorption and malnutrition or during remission phases, in cases of short bowels and/or high output intestinal stoma [5].

In patients with a stoma, the rapid gastric emptying, small bowel transit, and exclusion of the colon compromise the absorption of water, electrolytes, and/or macronutrients. If the condition is left untreated, undernutrition and/or dehydration will result. In these cases, parenteral nutrition represents a lifesaving therapy and contributes to the surgical restoration of bowel continuity, when indicated [5,10,11].

After the first basal evaluation, follow-up visits were performed at 1, 3, and 6 months of nutritional support. During the first months of observation in the analysis of our data, only an increase in body weight and BMI, but not a significant improvement in biochemical parameters (except for BChE), were observed in both groups; possibly due to the improved hydration status and consequent hemodilution [12].

On the other hand, the increased BChE levels after 1, 3, and 6 months of nutritional support testify to the recovery of nutritional status and liver protein synthesis. Several studies have shown that BChE levels reflect amino acidic substrate availability and, in the case of malnutrition (with or without inflammation), the impaired synthesis of visceral proteins; as well as reduced levels of other classical nutritional indicators (e.g., albumin, lymphocyte count, and cholesterol).

Malnutrition, a disease within the disease, may negatively influence a patient’s prognosis. To our knowledge, it has not been clearly demonstrated whether a good nutritional status can reduce the number of disease relapses. [5] Certainly, an adequate nutritional status improves a patient’s quality of life, improves asthenia, and safely prepares the patient for surgical intervention (in the form of bowel continuity restoration). In fact, surgical intestinal rehabilitation cannot be considered until the patient achieves nutritional repletion. [11] In our study, 8 out of 13 (61.5%) patients (4 with jejunostomies and 4 with ileostomies) reached surgical restoration of bowel continuity, a drastic improvement in quality of life, and weaning from parenteral nutrition.

Even with the several reported limits, to our best knowledge, the literature lacks studies with detailed step-by-step descriptions for nutritional intervention in CD patients.

According to the ESPEN guidelines, patients with CD are at risk and should be screened for malnutrition at the time of diagnosis and thereafter on a regular basis (grade of recommendation 3A) [7]. The required nutritional interventions for malnourished patients differ, according to their current nutritional status, inflammation score, and malabsorption degree. During the acute phase, patients usually present a loss of appetite, abdominal pain, malabsorption, diarrhea, and increased intestinal losses. The magnitude of malnutrition depends on the extent and location of the disease (ileal versus colonic) and, in particular, on the severity of the systemic inflammatory response associated with disease flares.

In patients with active CD, nutrition—particularly, enteral nutrition—can act as a primary therapy to treat intestinal inflammation, particularly in children and adolescents, and/or as a supportive therapy in the case of secondary malnutrition [6,13,14,15,16].

During remission, the most frequent nutritional abnormalities principally result from malabsorption for past intestinal resections, which differ according to the presence of a jejuno-/ileostomy or of the colon in continuity.

Despite the division in two groups (A and B), the patient characteristics remained quite heterogeneous; due to the small number of patients, further divisions seemed inconvenient.

Patients with the active disease received several treatments, ranging from corticosteroids to azathioprine to biological drugs. To our knowledge, the direct effects of specific drugs on nutritional parameters have not yet been fully described in the literature. On the other hand, the active disease influences nutritional parameters due to systemic inflammation, as well as nutrient and fluid malabsorption. Undoubtedly, disease treatment may also improve nutritional status by reducing inflammation and increasing nutrient and fluid absorption. In our opinion, etiological therapy and treatment for malnutrition are two inseparable entities.

## 6. Conclusions

The main purpose of this manuscript was to testify the global effectiveness of nutritional intervention in patients with CD, regardless of the activity and evolution of the disease.

With this study, we intend to motivate research into more efficient and effective nutritional therapeutic strategies for these patients in the future.

Unfortunately, our data represent the experience of a single specialized center for clinical and artificial nutrition. Further studies in more homogeneous groups of patients, clinical conditions, and pharmacological protocols could be useful in evaluating whether an adequate nutritional status favorably affects the clinical course of Crohn’s disease and/or the quality of life of these patients.

## Figures and Tables

**Table 1:** Baseline characteristics of 63 patients with Crohn’s disease (CD) divided into 2 groups: Group A, active disease (38 patients) and Group B, malnutrition/malabsorption due to intestinal resection (25 patients).

		GROUP A Active Disease Median (range)	GROUP B Malabsorption Median (range)
Patients	*n*	38	25
Age	years	41.5 (16–75)	35 (22–74)
Body Weight	Kg	55 (41–90)	52.3 (34–70)
BMI	Kg/m^2^	19.5 (15.1–31.1)	19.3 (14.2–26.2)
WL	%	12.3 (1.4–43.2)	21.4 (1.5–39.3)
Disease duration	months	5.9 (0.05–28.9)	10.6 (0.3–29.9)

BMI: body mass index. WL: weight loss (body weight loss in the last six months). No statistically significant differences between the 2 groups.

**Table 2:** Baseline characteristics and therapeutic intervention in 63 CD patients divided into group A (38 pts with active disease) and group B (25 pts with malabsorption due to intestinal resection).

	Group A Active Disease	Group B Malabsorption
Patients (*n*)	38 (19 M, 19 F)	25 (15 F, 10 F)
BMI < 18.5 kg/m^2^	12/38 (31.6%)	11/25 (44.0%)
WL ≥ 5–10%	8/38 (21.1%)	8/25 (32.0%)
Hb ≤ 10 g/dL	9/38 (23.7%)	6/25 (24.0%)
Lymph count < 1500/µL	17/38 (44.7%)	11/25 (44.0%)
Albumin < 3.5 g/dL	17/38 (44.7%)	8/25 (32.0%)
BChe < 5000 U/L	12/38 (31.6%)	12/25 (48.0%)
**Time from CD Diagnosis** (months)	median 5.8 (0.05–28.93)	median 10.6 (0.3–29.9)
**Type of Surgical Intervention**		
No resections	11 + 1 stricturoplastic	1/25 (4.0%)
Ileo-colic resections	15/38 (39.5%)	8/25 (32.0%)
Multiple resection	11/38 (28.9%)	18/25 (72.0%)
Presence of a stoma	4/38 (10.5%)	9/25 (36.0%)
Jejuno/Ileostomy	1/3	4/5
**Therapeutic Intervention**		
Dietetic indications	38/38 (100%)	25/25 (100%)
Oral supplements	21/38 (55.3%)	11/25 (44%)
Parenteral multivitamins and electrolytes	9/38 (23.7%)	0
Parenteral iron	0	9/25 (36.0%)
Peripheral parenteral nutrition	3/38 (7.9%)	3/25 (12.0%)
Central parenteral nutrition	4/38 (10.5%)	10/25 (4%)

BMI: body mass index; BW loss (body weight loss in the last six months); Hb: Hemoglobin. Lymph count: lymphocyte count; BChe: butyryl-cholinesterase. WL: Weight Loss (body weight loss in the last six months); M: males; F: females, CD: Crohn’s Disease.

**Table 3:** Anthropometric parameters and biochemical data of 38 patients with acute Crohn’s disease evaluated at first visit and after 1, 3, and 6 months of nutritional rehabilitation.

		Baseline Mean ± SD	1 Month Mean ± SD	3 Months Mean ± SD	6 Months Mean ± SD
Body Weight	Kg	58.4 ± 12.2	59.7 ± 10.6	60.4 ± 11.3 **	61.3 ± 8.4
BMI	Kg/m^2^	20.7 ± 4,3	21.2 ± 3.2	22.7 ± 3.3 **	23.1 ± 3.8
Hb	g/dL	12.2 ± 1.6	12.4 ± 1.2	12.6 ± 2.1 **	13.5 ± 1.6 ***
Lymph count	n/µL	1680 ± 542	1711 ± 628	1616 ± 947	1786 ± 665
Transferrin	mg/dL	2.1 ± 0.4	2.4 ± 0.5	2.4 ± 0.4	2.5 ± 0.9
Albumin	g/dL	3.5 ± 0.7	3.6 ± 0.6	3.7 ± 0.5	3.8 ± 0.5
Prealbumin	g/dL	0.20 ± 0.07	0.24± 0.1	0.22 ± 0.06	0.24 ± 0.03
Total Cholesterol	mg/dL	125 ± 35	133 ± 46	147 ± 49	156 ± 40 *
BChE	UI/mL	5854 ± 1981	6944 ± 2817 *	7854 ± 2718 *	10,959 ± 2671 */**/***

BMI: body mass index; WL (body weight loss in the last 6 months); Hb: hemoglobin; Lymph count: lymphocyte count; BChE: butyrrylcholinesterase. * *p* < 0.05 compared with baseline; ** *p* < 0.05 compared with 1 month; *** *p* < 0.05 compared with 3 months.

**Table 4:** Anthropometric parameters and biochemical data of 25 patients with malabsorption due to past intestinal resection for Crohn’s disease evaluated at first visit and after 1, 3, and 6 months of nutritional rehabilitation.

		Baseline Mean ± SD	1 Month Mean ± SD	3 Months Mean ± SD	6 Months Mean ± SD
Body Weight	Kg	53.1 ± 15.5	56.3 ± 12.0 *	59.1 ± 12.1 **	62.1 ± 12.3 ***
BMI	Kg/m^2^	19.6 ± 3.4	20.9 ± 3.4 *	21.5 ± 30.4 **	22.7 ± 4.1 ***
Hb	g/dL	11.6 ± 1.9	11.5 ± 1.7	11.9 ± 1.5 **	12.6 ± 1.5 ***
Lymph count	n/µL	1765 ± 1233	1711 ± 1097	1705 ± 837	1572 ± 917
Transferrin	mg/dL	2.6 ± 0.7	2.7 ± 0.64	3.06 ± 0.4	2.7 ± 0.6
Albumin	g/dL	3.8 ± 0.9	3.7 ± 0.8	3.9 ± 1.1	3.8 ± 0.9
Prealbumin	g/dL	0.21 ± 0.07	0.26 ± 0.03	0.22 ± 0.06	0.27 ± 0.03
Total Cholesterol	mg/dL	146 ± 34	144 ± 31	159 ± 34 *	165 ± 35 *
BChE	UI/mL	6275 ± 1701	6939 ± 1876 *	7845 ± 1978*/**	8554 ± 2158 */**

* *p* < 0.05 compared with baseline; ** *p* < 0.05 compared with 1 month; *** *p* < 0.05 compared with 3 months.
